# Impact of SHP2 tyrosine phosphorylation on the development of acquired resistance to allosteric SHP2 inhibitors

**DOI:** 10.18632/oncotarget.28392

**Published:** 2023-03-31

**Authors:** Giulia Franciosa, Jesper V. Olsen

**Keywords:** SHP2, PTPN11, tyrosine, phosphorylation

The SH2 domain-containing tyrosine phosphatase 2 (SHP2) is a ubiquitously expressed non-receptor protein tyrosine phosphatase, encoded by the *PTPN11* gene. It is positively regulated by upstream receptor tyrosine kinases (RTKs) to activate downstream the RAS-ERK pathway [1].

AML is a bone marrow malignancy characterized by a blockage of differentiation and an uncontrolled proliferation of myeloid hematopoietic progenitor cells. The internal tandem duplication (ITD) in the juxta-membrane domain of the RTK FLT3 is an oncogenic driver mutation that leads to constitutive activation of its tyrosine kinase activity. Consequently, FLT3 inhibitors that block its tyrosine kinase activity represent the targeted treatment option for patients with FLT3-ITD AML, often administrated in combination with induction chemotherapy. Nevertheless, the short duration of remission urges the development of novel combinatorial therapies for FLT3-ITD AML [2].

Since 2016, several potent and selective allosteric, noncovalent SHP2 inhibitors have been developed and tested in clinical trials for solid tumors [3]. A recent study reported the effectiveness of short-term treatment with the allosteric SHP2 inhibitor SHP099 as a single agent in clinically relevant mouse models of Flt3-ITD AML [4]. This observation was in contrast with published data showing that allosteric SHP2 inhibition is only effective as combination treatment with inhibitors of other nodes of the RAS-ERK pathway [5].

In a research article published in Cancer Research by Pfeiffer et al. [6], the Olsen’s lab at University of Copenhagen showed that two commercial FLT3-ITD-positive AML cell lines (MV-4-11 and MOLM-13) developed adaptive resistance after prolonged treatment *in vitro* with the allosteric SHP2 inhibitor SHP099.

To identify the global molecular changes induced by resistance to SHP099, they employed quantitative mass spectrometry (MS)-based proteomics and phosphoproteomics, which represents the most comprehensive approach for quantitative profiling of proteins and their post-translational modifications (PTMs) [7]. They observed a strong dephosphorylation of ERK1 and ERK2 (pERK) after short-term SHP099 treatment, and its return to baseline level of parental cell lines after longer time points and in resistant cells. ERK re-phosphorylation was MEK-dependent, as it was prevented by MEK inhibition. The authors also observed regulation of the Tyr-542 of SHP2 following the dynamics of pERK, suggesting direct involvement of SHP2 in pERK dynamics. To test this hypothesis, the authors performed double SHP2 inhibition with SHP099 and the active site inhibitor II-B08 or SHP2 siRNA-based knockdown, showing that pERK rebound was dependent on SHP2 phosphatase activity.

**Figure 1:**
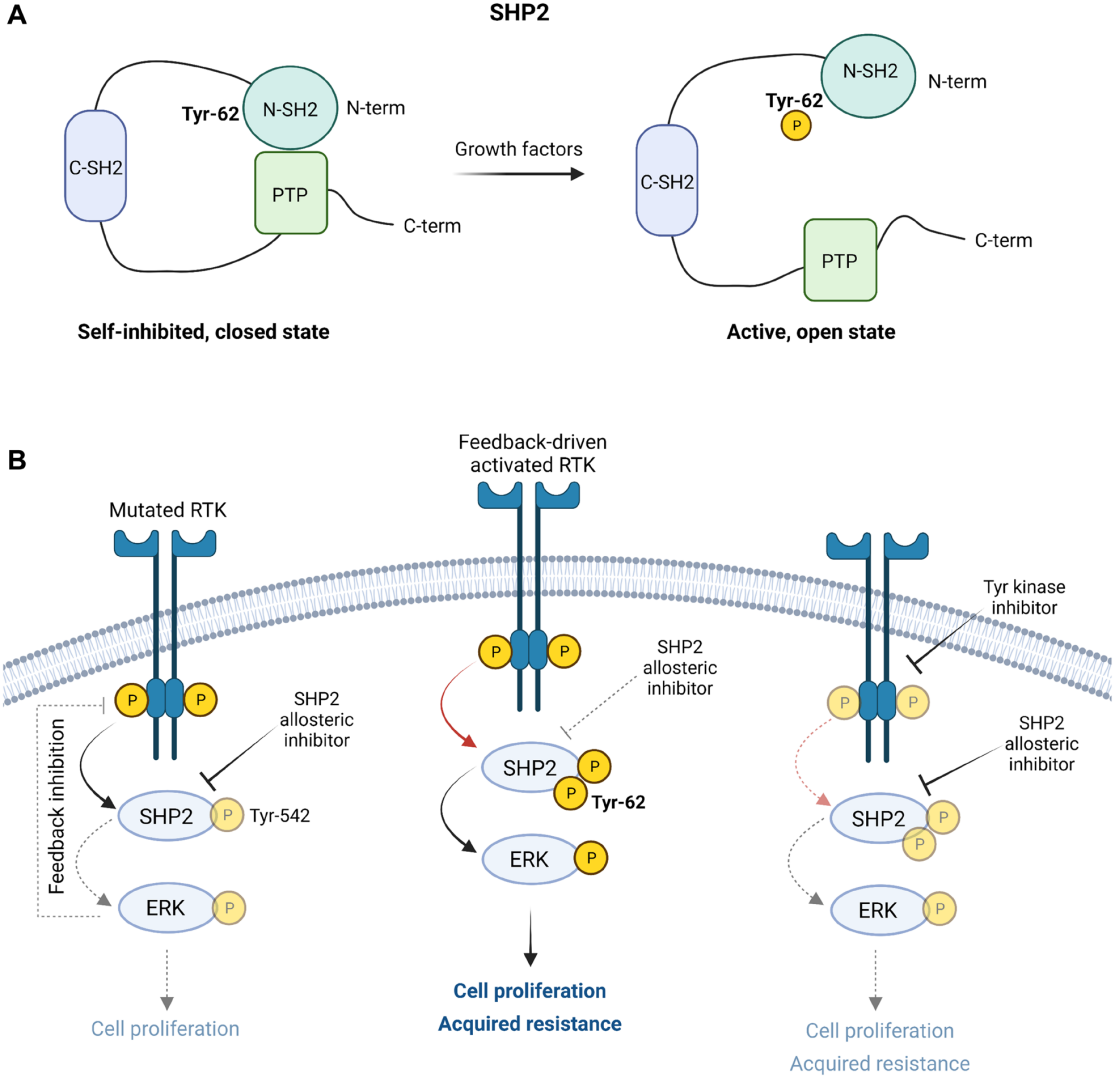
Graphical illustration of the main findings from Pfeiffer et al., Cancer Research, 2022. (**A**) Role of Tyr-62 phosphorylation in SHP2 activation. When Tyr-62 is phosphorylated (e.g., in presence of growth factors), the intramolecular interactions between the N-terminal Src Homology 2 (N-SH2) and the Protein-tyrosine phosphatase (PTP) domains of SHP2 are lost, causing the exchange between its self-inhibited/close state to its active/open state. (**B**) AML and B-ALL cells harboring activating mutations in either FLT3 or KIT receptor tyrosine kinases (RTKs) develop acquired resistance to allosteric SHP2 inhibitors, like SHP099, by upregulating the RTK-SHP2-ERK axis through phosphorylation of Tyr-62 on SHP2. Resistance can be reversed by combined treatment with RTK inhibitors. Created with https://www.biorender.com/.

Next, the researchers postulated that SHP099 was not able to bind its target in acquired resistant cells. To sustain this hypothesis, they identified the Tyr-62 on SHP2 displaying a specific dynamic behavior characterized by an absence of regulation upon SHP099 treatment in parental cells and an increase only in resistant cells. This site is part of the N-terminal Src Homology 2 (SH2) domain of SHP2 (Figure 1A), which is a known mutational hotspot in multiple diseases, including Noonan syndrome and Juvenile Myelomonocytic Leukemia (JMML). Mutations in this region generally result in constitutively active SHP2 mutants by stabilizing the open conformation of SHP2, thus antagonizing SHP2 inhibition by SHP099 and other allosteric inhibitors that act by maintaining the self-inhibited conformation [8]. In support of this hypothesis, the authors showed that wild-type and non-phosphorylatable SHP2 mutant (Y62F) could bind to SHP099, whereas the phosphomimetic form (Y62E) could not. This proved that Tyr-62 is responsible for the ineffectiveness of SHP099 in inhibiting SHP2 activity through stabilization of SHP2 in its open, active conformation (Figure 1A).

The researchers also uncovered that FLT3 was itself regulated on its autophosphorylation site Tyr-969, following the same phosphorylation site regulation pattern of SHP2 Tyr-62, suggesting that FLT3 was the RTK responsible for SHP2 phosphorylation and reactivation. This hypothesis was supported by results showing that the combination of SHP2 allosteric inhibitors and second generation FLT3 inhibitors had a synergistic effect in reducing reactivation of ERK and the survival of resistant cells.

The researchers were able to validate these findings in two other mutated RTK-driven leukemia models: the B-ALL cell line HB11;19 and the inv (16)/Kit^D816Y^ AML mouse model. HB11;19 harbors the FLT3 point mutation D835H, located in its tyrosine kinase domain, leading to ligand independent FLT3 kinase activation. The inv (16)/Kit^D816Y^ AML mouse model expresses the RTK Kit with the oncogenic mutation KitD816Y, which causes constitutive activation of the receptor.

In summary, this study reveals a molecular escape mechanism of mutated RTK-driven leukemic cells from SHP2 allosteric inhibition, relying on a non-genetic on target reactivation. Mechanistically, SHP2 inhibition induces tyrosine phosphorylation and feedback-driven activation of the mutated RTK, which in turn phosphorylates SHP2 on Tyr-62. This phosphorylation confers resistance by preventing allosteric inhibitors binding to SHP2 (Figure 1B). Further research is warranted to investigate whether acquired resistance arises in tumors other than AML and B-ALL.

All in all, the findings by Pfeiffer et al. suggest that combined inhibition of SHP2 and mutated RTKs are effective in preventing adaptive resistance, but also highlight the need for development of more potent and effective SHP2 inhibitors and combination therapies for clinical applications.
